# Correlations between weight perception and overt risk-taking among Canadian adolescents

**DOI:** 10.17269/s41997-023-00778-1

**Published:** 2023-06-23

**Authors:** Sydney Bartlett, Jana Bataineh, Wendy Thompson, William Pickett

**Affiliations:** 1 Department of Health Sciences, Brock University, St. Catharines, ON Canada; 2 Centre for Surveillance of Applied Research, Public Health Agency of Canada, Ottawa, ON Canada; 3 Department of Public Health Sciences, Queen’s University, Kingston, ON Canada

**Keywords:** Weight perception, Risk-taking, Canadian, Adolescents, HBSC, Perception du poids, prise de risques, Canada, adolescent, Enquête HBSC

## Abstract

**Objective:**

Perceptions of body weight represent an important health issue for Canadian adolescents. While associations between weight perception and mental health concerns like eating disorder symptomatology are well established, there is need for more Canadian evidence about how weight perception is associated with overt risk-taking among adolescents, and further how such associations differ by biological sex.

**Methods:**

We conducted a national analysis of grade 9–10 students participating in the 2017–2018 cycle of the Health Behaviour in School-aged Children (HBSC) study in Canada. This analysis described contemporary patterns of alternate weight perception and then examined the strength and statistical significance of such associations by biological sex, with tobacco, alcohol, and cannabis use, binge drinking, fighting, and illicit drug use as outcomes. Behaviours were considered both individually and in combination. Analyses were descriptive and analytical, with regression models accounting for the nested and clustered nature of the sampling approach.

**Results:**

Responses from 2135 males and 2519 females were available for a complete case series analysis. A total of 26% and 35% of males and females, respectively, perceived themselves as “too fat” while 20% and 9% identified as “too thin”. Females perceiving themselves as “too fat” reported higher likelihoods of engaging in individual and scaled indicators of overt risk-taking. Conversely, among males, alternate weight perception was associated with lower levels of such behaviours.

**Conclusion:**

As males and females perceive and react to weight perception differently, clinical and health promotion strategies should be developed and uniquely targeted to groups of adolescents in regards to weight perception and risk-taking.

## Résumé

**Objectif:**

Les perceptions du poids corporel représentent une importante question de santé pour les adolescentes et les adolescents canadiens. Les associations entre la perception du poids et les risques pour la santé mentale, comme les symptômes de troubles alimentaires, sont bien établies, mais nous avons besoin de plus de données probantes canadiennes sur les associations entre la perception du poids et la prise de risques manifeste chez les adolescentes et les adolescents, et sur les différences selon le sexe biologique lorsque ces associations existent.

**Méthode:**

Nous avons mené une analyse nationale des élèves de 9e et de 10e année ayant participé au cycle 2017-2018 de l’Enquête sur les comportements de santé des jeunes d’âge scolaire au Canada (Enquête HBSC). Nous avons décrit les tendances contemporaines de perception erronée du poids, puis examiné la force et la signification statistique de telles associations, selon le sexe biologique, avec la consommation de tabac, d’alcool et de cannabis, l’hyperalcoolisation rapide, les combats et l’usage de drogues illicites comme résultats. Ces comportements ont été étudiés individuellement et en combinaison. Les analyses ont été descriptives et analytiques, avec des modèles de régression pour tenir compte de la méthode d’échantillonnage emboîtée et par grappes.

**Résultats:**

Les réponses de 2 135 garçons et de 2 519 filles étaient disponibles pour une analyse complète de série de cas. En tout, 26 % des garçons et 35 % des filles se percevaient comme étant « en excès de poids », et 20 % des garçons et 9 % des filles se percevaient comme étant « trop maigres ». Les filles se percevant comme étant « en excès de poids » ont déclaré une probabilité plus élevée d’adopter des comportements individuels et gradués indicateurs d’une prise de risques manifeste. Réciproquement, chez les garçons, la perception erronée du poids était associée à des niveaux moins élevés de tels comportements.

**Conclusion:**

Comme les garçons et les filles perçoivent leur poids différemment et réagissent différemment à cette perception, il faudrait élaborer des stratégies cliniques et de promotion de la santé qui ciblent particulièrement les groupes d’adolescentes et d’adolescents et qui portent sur la perception du poids et la prise de risques.

## Introduction

Weight perception describes how one perceives their weight, and by extension their physical appearance (Rawana, 2013). It is common for adolescents with alternate weight perception (perceiving themselves to be either “too thin” or “too fat”) to also struggle with low body-satisfaction and consequently suffer from negative mental health outcomes (Rawana, 2013). This issue is especially pronounced in populations of young people, with a growing number of adolescents reporting some level of dissatisfaction with their physical appearance (Liu et al., 2019). Being dissatisfied with one’s body is linked to several negative health outcomes along the life course, such as self-harm and cannabis, tobacco, and alcohol use (Bornioli et al., 2019). Alternate weight perception has also been associated with binge drinking and breakfast skipping (Raffoul et al., 2018b). Weight perception and body-satisfaction are related constructs, and it is important to explore how alternate weight perception is related to other factors that influence health status. Externalizing symptoms of mental health challenges, including risk-taking, are one such group of behaviours.

Weight perception may be expressed differently in males and females (Deschamps et al., 2014), just as the societal standards for appearance vary by biological sex (Liu et al., 2019). Males are more likely to feel pressured to satisfy society’s ideals for musculature, whereas females are more likely to strive to be smaller to satisfy societal pressures for petiteness (Liu et al., 2019). A body of literature shows that alternate weight perception can result in a variety of negative internalized feelings and emotions (Rawana, 2013), and eating disorder symptomatology (Deschamps et al., 2014). Less is known, however, about whether externalized aspects of mental health, such as overt risk-taking, relate strongly to alternate weight perception. While there is evidence that alternate weight perception is associated with increased binge drinking (Raffoul et al., 2018b), increased smoking frequency (Yoon and Bernell, 2016), and symptoms of depression (Rawana, 2013) and anxiety (Isomaa et al., 2011), these variables are often examined in isolation. Similarly, fewer related studies have been conducted with male adolescents, with analyses tending to be restricted to females. Exploration of these associations is important; if unresolved, and no matter what the cause, overt risk-taking that emerges during adolescence can be part of negative life course trajectories that are associated with elevated risks for injury, suicide, and chronic disease (Ammerman et al., 2016).

Biological and social theories are germane to this field of study. Alternate weight perception is linked with both anxiety and distress, depressive symptoms, and low self-esteem (Isomaa et al., 2011). In turn, these feelings have physiological consequences (e.g., excess cortisol production) which can lead to a propensity to take risks (Armstrong-Carter and Telzer, 2022). From a psychological perspective, coping theory (Stallman, 2021) is helpful interpretively. Under this theory, adolescents may engage in risk-taking for several reasons, including to relate to peers, to deal with negative feelings or to feel a sense of belonging in a social group (Stallman, 2021; Pound & Campbell, 2015; Sharma & Morrow, 2016). An adolescent’s developing brain can lead to decision-making that is based upon whim rather than reason (Institute of Medicine, 2011). Hypothetically, this explains why an adolescent may choose to cope by taking risks in response to the emotional turmoil that accompanies alternate weight perception (Isomaa et al., 2011). Therefore, as alternate weight perception can theoretically trigger overt risk-taking, it is important for public health professionals to consider this risk-taking catalyst when developing interventions. Previous research has examined clustered risk-taking in the form of breakfast skipping, tobacco usage, and binge drinking among Canadian females who had reported alternate weight perception (Raffoul et al., 2018a). We would like to build upon such analyses by examining additional risk-taking behaviours and conducting a sex-stratified analysis, including both males and females.

Utilizing a contemporary national sample of young Canadians and informed by the above theories, we quantified the strength and consistency of associations between weight perception and risks for engagement in individual and clustered risk-taking behaviours among male and female adolescents. Our hope was that our analyses would provide new evidence on the potential consequences of alternate weight perception in young people, and to understand more about the potential etiology of overt risk-taking in these same populations.

## Methods

### Study base

**Fig. 1:**
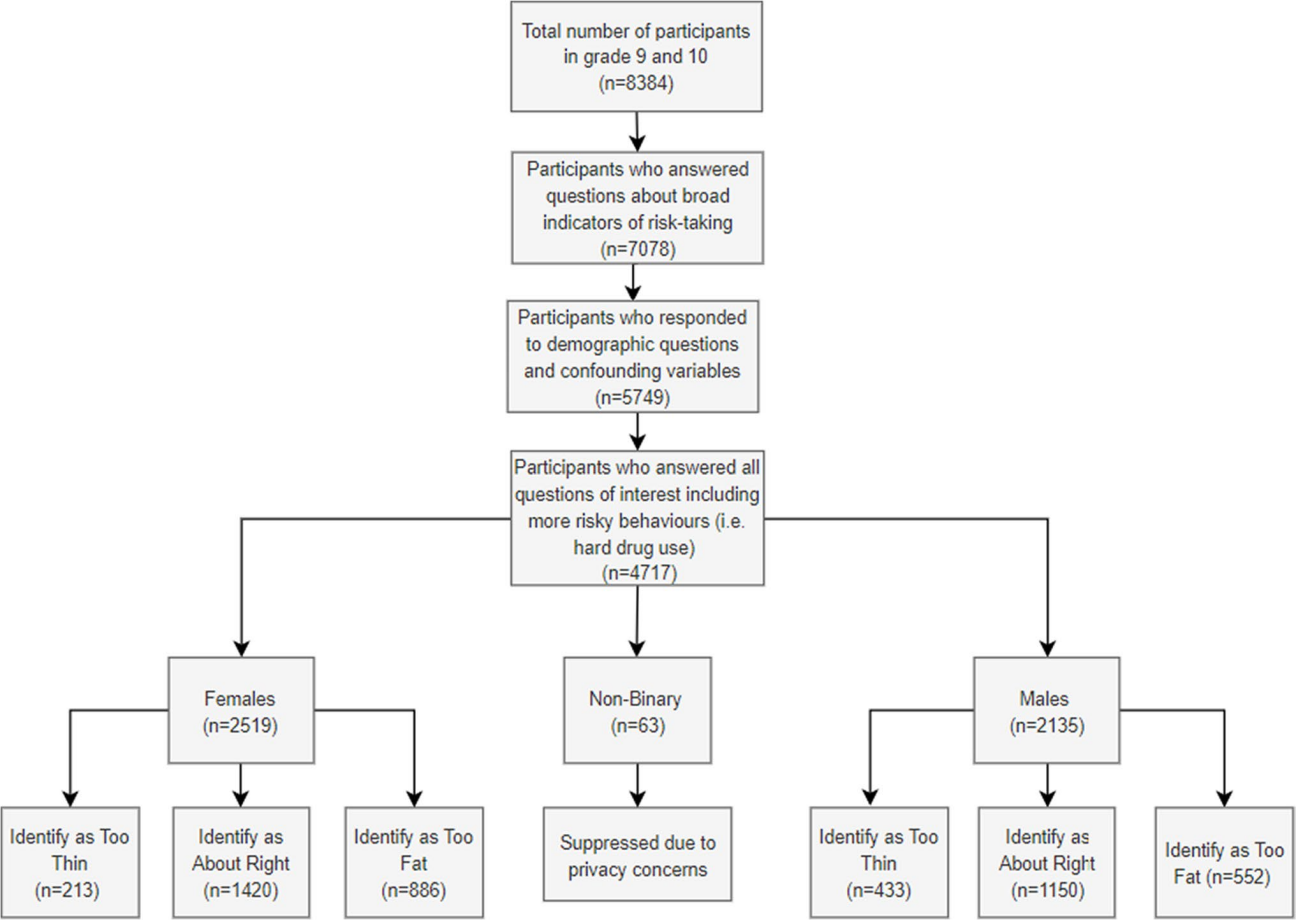
Flow chart describing how the final sample was collected; of 8384 participants, 7078 responded to broad indicators of risk-taking, 5749 completed items for all demographic questions and confounding variables, and 4717 completed all variables of interest including higher risk behaviours (i.e., hard drug use), 2519 of whom were female, 2135 of whom were male, and 63 of whom were non-binary. Among females, 213 identified as “too thin”, 1420 identified as “about right”, and 886 identified as “too fat”. Among males, 433 identified as “too thin”, 1150 identified as “about right”, and 552 identified as “too fat”. The non-binary sample was supressed due to privacy concerns

Data used in this analysis were from Cycle 8 (2017/2018) of the Health Behaviour in School-aged Children Survey (HBSC) (Craig et al., 2017). The HBSC aims to “gain insight and increase understanding of health and its determinants of young people via a cross-sectional survey administered to students every four years” (Craig et al., 2017). For this analysis, we accessed reports from 8384 Canadian students from across the country who completed the grade 9–10 questionnaire (Craig et al., 2017). Further inclusions were as follows: (1) complete information on key variables of interest; (2) provision of informed consent; and (3) self-identification as male or as female, leading to a final sample of 4654 students (2519 females and 2135 males) (Fig. 1). Although some of this decrease in sample size can be attributed to non-response, it can mainly be attributed to the variables of interest not being asked in all age groups or across all provinces and territories in Canada. Some jurisdictions administered shortened versions of the HBSC questionnaire to accommodate varying levels of literacy and appropriateness for different stages of development, as well as the preferences of school administrations.

### Ethical considerations

HBSC holds ethics approval from the General Research Ethics Board at Queen’s University, as well as the Public Health Agency of Canada Research Ethics Board/Health Canada. This secondary data analysis was reviewed and approved by the Research Ethics Board at Brock University.

### Key variables

#### Weight perception (primary exposure)

Participants were asked to categorize their feelings toward their body size into one of the following categories: “much too thin”, “too thin”, “about the right size”, “too fat”, and “much too fat” (Craig et al., 2017). These variables were further categorized into “too thin”, “about the right size”, and “too fat” for the main analysis. The “much too fat” variable was also explored for females. Alternate weight perception refers to the response categories that were not “about the right size”.

#### Risk-taking (primary outcome)

Participants described their engagement (yes or no, or level of engagement) in various types of risk-taking using specific items. *Tobacco use* was evaluated based upon indications of whether each student reported that they had, or had not, smoked or used each of cigarillos, cigars, e-cigarettes, chew, or nicotine in the past 30 days (Craig et al., 2017). These variables were examined both individually and in composite. Additional variables were used to describe “ever” or “never” engagement in risk-taking behaviours, i.e., *cannabis use* (last 30 days); *binge drinking* (5 or more drinks on one occasion for males, 4 or more for females in the last 30 days) (Centre for Addiction and Mental Health, 2022); *physical fighting* (last 12 months) (Pickett et al., 2005); and *drug use* of any of several types of substances (ecstasy, amphetamines, methamphetamines, heroin, cocaine, glue, LSD and hallucinogens, pain killers, stimulants, sedatives, and cough medicines “to get high” in the last 12 months) (Craig et al., 2017).

We also measured four types of risk-taking in composite measures, as per precedent (Kwong et al., 2017). These included scales in three domains describing (1) tobacco use; (2) cannabis or alcohol use; and (3) use of prescription medications or illicit drugs to get high. For each domain, we classified behaviours into three groups (none, low, high). The three domains were also combined to create an *overall risk-taking score* that ranged between 3 (no engagement in any domain of risk behaviour) and 9 (maximum engagement in all behaviours).

#### Sex (stratification variable)

Participants were asked to identify themselves as either “male”, “female” or “neither term describes me” (i.e., non-binary identity). While this variable is sometimes used to infer a young person’s gender identity, after exclusion of the non-binary students for privacy reasons (the potential for re-identification), here the male or female designation most appropriately is interpreted as an indicator of biological sex.

#### Potential confounders

Participants identified at least one of fourteen *racial/ethnic* categories, which were in turn used to classify them into ethnic status groups. Participants could identify as White, Chinese, South Asian, Black, Filipino, Latin American, Southeast Asian, Arab, Japanese, Korean, First Nations, Metis, Inuit, or Other (including mixed race). Due to sample size concerns, these groups had to be combined into two categories, White and non-White, to complete the data analysis. Participants also rated “how well off they think their family is” (*5 socioeconomic categories* from “very well off” to “not at all well off”) (Currie et al., 2014). *Geographic location* was inferred from school addresses, and available indicators used province/territory and the population size and density of the school community, measured using Statistics Canada criteria (Statistics Canada, 2016). *Immigration status* was inferred from a single indicator (“born in Canada”, “lived in Canada 1–2 years”, “lived in Canada 3–5 years”, “lived in Canada 6–11 years”, and “lived in Canada 11 or more years”) (Kukaswadia et al., 2014). Finally, *eating habits* (one indicator of relative healthfulness) were estimated based upon a brief, 4-item food frequency questionnaire (Vereecken & Maes, 2003). The 4 items included frequency of fruit, vegetable, sweet, and soft drink consumption. Fruits and vegetables were grouped into a “healthy food” index while the sweets and soft drinks were grouped into an “unhealthy food” index.

### Statistical analysis

The prevalence and patterns of alternate weight perception and overt risk-taking (both overall and for specific behaviours) were described by sex and other sociodemographic factors. Multivariable logistic regression analyses, stratified by sex, were used to estimate the strength and statistical significance of associations between several categories of alternate weight perception using “about right” as the referent group, and engagement in risk-taking, as measured by our primary composite scale. A backwards elimination process (American Psychological Association (APA), 2020) followed by change in estimation methods (Talbot et al., 2021) were used to develop parsimonious models. Effects were summarized as crude and adjusted odds ratios at the 95% significance level, with standard errors adjusted for clustering, using an estimated design effect of 1.04. All analyses were conducted in RStudio version 2021.09.1 + 372 (RStudio Team, 2020).

## Results

Table 1 describes the study population demographically by sex, then by weight perception and various indicators of overt risk-taking. Alternate weight perception was experienced differently between males and females and was also related to different likelihoods of engaging in overt risk-taking. Females were more likely to self-identify as “too fat” (35%), and quite unlikely to identify as too thin (9%). Compared to females, fewer males reported that they were too fat (26%) and higher proportions described their body as being “too thin” (20%). Engagement in overt risk-taking was also common among both sexes, with similar percentages of males and females reporting moderate to high levels of engagement, in both individual and scaled measures.

**Table 1:** Descriptive factors of the study population (*N* = 4654), as well as the proportion of participants who experienced the exposure and the outcome of interest*, stratified by sex

	Males (*n* = 2135)		Females (*n* = 2519)	
	*n*	% total	*n*	% total
Study population				
Race				
White	1506	70.5	1790	71.1
Minority group	629	29.5	729	28.9
Immigration status				
Canadian-born	1614	75.6	2030	80.6
Immigrant	521	24.4	489	19.4
Urban status				
Rural	1054	49.3	1201	47.7
Medium-sized urban centre	463	21.7	542	21.5
Large urban centre	618	28.9	776	30.8
Socioeconomic status				
Low	712	33.3	918	36.4
Medium	779	36.5	911	36.2
High	644	30.2	690	27.4
Primary exposure				
Weight perception				
About right	1150	53.9	1420	56.4
Too thin	433	20.3	213	8.5
Too fat	552	25.6	886	35.2
Primary outcomes				
Level of risk-taking*				
Medium	756	35.4	925	36.7
High	345	16.2	389	15.4
Tobacco use				
Medium	425	19.9	494	19.6
High	149	7.0	108	4.3
Alcohol and cannabis use				
Medium	521	24.4	659	26.2
High	295	13.8	351	13.9
Illicit and prescription drug use				
Medium	275	12.9	356	14.1
High	133	6.2	153	6.0

*Levels of risk-taking as determined by our composite scale

Among females, self-identification as “too fat” was associated with increased odds of engaging in higher levels of risk-taking (OR = 1.28, 95% CI 1.03–1.63) (Table 2). Similar results were seen in terms of individual substance use behaviours, such as alcohol or cannabis use (OR = 1.30, CI: 1.08–1.56) and use of tobacco products (OR = 1.28, CI: 1.04–1.57). An even more substantial association was observed for females who identified as “much too fat”, with OR = 2.30, CI: 1.50–3.51 for moderate to high levels of risk-taking (Table 3); OR = 2.44, CI: 1.58–3.75 for use of tobacco products; and OR = 1.73, CI: 1.12–2.65 for alcohol or cannabis consumption (Table 2). Among females who identified as being “too thin”, associations were less consistent, with only the relationship between risk-taking and alcohol or cannabis consumption reaching significance (OR = 1.36, CI: 1.00–1.84). Crude odds ratios are also presented in Tables 2 and 3.

**Table 2:** Results of logistic regression analysis: risk of different outcomes of interest, associated with weight perception, crude, and adjusted* analyses, in females

	Outcome	Crude		Fully adjusted	
	% total	OR (95% CI)		OR (95% CI)	
Domain 1: Tobacco use					
About right	21.5	1.00	Referent	1.00	Referent
Too thin	25.3	1.24	0.88 to 1.72	1.18	0.83 to 1.69
Too fat	27.3	1.37	1.13 to 1.66	1.28	1.04 to 1.57
Much too fat	43.1	2.75	1.84 to 4.10	2.44	1.58 to 3.75
Domain 2: Cannabis and alcohol use					
About right	37.1	1.00	Referent	1.00	Referent
Too thin	43.7	1.31	0.97 to 1.76	1.36	1.00 to 1.84
Too fat	44.0	1.22	1.12 to 1.58	1.30	1.08 to 1.56
Much too fat	52.3	1.86	1.26 to 2.75	1.73	1.12 to 2.65
Domain 3: Prescription and illicit drug use					
About right	21.0	1.00	Referent	1.00	Referent
Too thin	21.1	1.01	0.70 to 1.42	1.00	0.69 to 1.45
Too fat	18.7	0.87	0.70 to 1.07	0.85	0.68 to 1.06
High risk					
About right	13.6	1.00	Referent	1.00	Referent
Too thin	16.9	1.29	0.86 to 1.94	1.26	0.84 to 1.87
Too fat	18.1	1.40	1.10 to 1.78	1.28	1.03 to 1.63
Binge drinking					
About right	28.9	1.00	Referent	1.00	Referent
Too thin	32.9	1.21	0.88 to 1.63	1.20	0.86 to 1.68
Too fat	33.1	1.22	1.01 to 1.47	1.17	0.96 to 1.43
Physical fighting					
About right	12.5	1.00	Referent	1.00	Referent
Too thin	17.4	1.47	0.98 to 2.14	1.30	0.86 to 1.97
Too fat	17.8	1.47	1.15 to 1.87	1.33	1.03 to 1.71

*Results were adjusted for SES, race, immigration status, diet, and urban status

**Table 3:** Results of logistic regression analysis: risk of moderate to high risk-taking associated with weight perception, crude, and adjusted* analyses, in males and females

	Moderate to high levels of overt risk-taking					
Weight perception	Total	% outcome	Crude OR (95% CI)		Adj OR (95% CI)	
Males						
Too thin	433	28.2	0.87	(0.68 to 1.13)	0.84	(0.65 to 1.10)
About right	1150	31.0	1.00	Referent	1.00	Referent
Too fat	552	30.1	0.96	(0.77 to 1.22)	0.94	(0.74 to 1.20)
Females						
Too thin	213	32.8	1.20	(0.87 to 1.66)	1.20	(0.86-1.67)
About right	1420	28.9	1.00	Referent	1.00	Referent
Too fat	886	33.8	1.25	(1.04 to 1.52)	1.21	(1.00 to 1.47)
Much too fat	109**	50.5	2.50	(1.66 to 3.76)	2.30	(1.50 to 3.51)

*Results were adjusted for SES, race, immigration status, diet, and urban status

**The females counted as “much too fat” were included into the “too fat” group, but also investigated separately

Among males (Table 4), very different findings were observed. Males who self-identified as “too thin” were significantly less likely to participate in binge drinking (OR = 0.67, CI = 0.51 to 0.88) relative to males who perceived themselves to be the right size. Males who identified as “too fat” were also at a lower risk of binge drinking (OR = 0.70, CI: 0.62 to 1.00) relative to males who identified as “about the right size”. Crude odds ratios are also presented in Tables 3 and 4.

**Table 4:** Results of logistic regression analysis: risk of different outcomes of interest, associated with weight perception, crude, and adjusted* analyses, in males

	Outcome	Crude		Fully adjusted	
	% total	OR (95% CI)		OR (95% CI)	
Domain 1: Tobacco use					
About right	26.5	1.00	Referent	1.00	Referent
Too thin	28.6	1.11	0.86 to 1.44	1.09	0.84 to 1.43
Too fat	26.3	0.98	0.78 to 1.25	0.95	0.74 to 1.22
Domain 2: Cannabis and alcohol use					
About right	40.3	1.00	Referent	1.00	Referent
Too thin	34.6	0.79	0.62 to 1.00	0.77	0.60 to 0.99
Too fat	36.8	0.86	0.69 to 1.07	0.85	0.67 to 1.06
Domain 3: Prescription and illicit drug use					
About right	19.1	1.00	Referent	1.00	Referent
Too thin	20.1	1.06	0.80 to 1.42	1.02	0.76 to 1.36
Too fat	18.3	0.95	0.72 to 1.24	0.92	0.70 to 1.22
High risk					
About right	16.5	1.00	Referent	1.00	Referent
Too thin	17.6	1.07	0.79 to 1.45	0.99	0.72 to 1.37
Too fat	14.3	0.84	0.63 to 1.13	0.77	0.57 to 1.05
Binge drinking					
About right	32.6	1.00	Referent	1.00	Referent
Too thin	24.9	0.67	0.53 to 0.89	0.67	0.51 to 0.88
Too fat	28.3	0.81	0.65 to 1.03	0.70	0.62 to 1.00
Physical fighting					
About right	31.0	1.00	Referent	1.00	Referent
Too thin	28.2	0.87	0.68 to 1.12	0.82	0.63 to 1.06
Too fat	29.8	0.95	0.75 to 1.19	0.91	0.72 to 1.15

*Results were adjusted for SES, race, immigration status, diet, and urban status

## Discussion 

In this analysis, we explored weight perception among young people in Canada and how it related to engagement in overt risk-taking. A priori we hypothesized that males and females would experience such perceptions and their potential effects differently. Consistent with expectations (Liu et al., 2019), females were more likely to report feeling that their body was “too fat”, while males were more likely to report that their body was “too thin”. Females who perceived their body to be “too fat” were significantly more likely to smoke, use cannabis or alcohol, engage in physical fighting, and engage in high levels of overt risk-taking. In contrast, males more often felt that their body was “too thin”, and these perceptions were generally associated with lower levels of engagement in overt risk-taking both measured in composite and in terms of individual behaviours.

Our findings can be framed in the context of coping theory (Stallman, 2021). As adolescents deal with the perceived stigma and internalized feelings associated with alternate weight perception, they may resort to risk-taking behaviours to cope (Stallman, 2021). Risk-taking can relieve the distress that they may be facing through several mechanisms. First, it may serve as a “rite of passage” into a social group, leading to a feeling of group acceptance, despite their perception of incongruence with societal body ideals (Pound & Campbell, 2015). Substance use is also recognized as a coping mechanism that some adolescents lean towards when dealing with negative emotions, like those brought on by alternate weight perception (Sharma & Morrow, 2016). This is consistent with our results, which show that females who perceive themselves as “too fat” have higher likelihoods of engagement with alcohol, cannabis, and tobacco products, both alone and in combination. Furthermore, some risk-taking behaviours can allow adolescents to cope by directly addressing the perceived issues with their body. For example, the nicotine which is found in many tobacco products is an appetite suppressant and has been previously identified as a weight-control tactic among females (White, 2012).

Such theories might be further interpreted with consideration of the influence of biological sex. For example, females may be especially prone to perceiving social approval from peers when they engage in risk-taking behaviours. In addition, females who report alternate weight perception are known to perceive disapproval from family and community based on their bodies; hence, further adult disapproval with respect to their risk-taking may be of less concern to them (Nishida et al., 2019). Among males with alternate weight perception, the opposite trend occurs where the avoidance of risk-taking becomes more characteristic. Existing cognitive models suggest that males with alternate weight perception may be too embarrassed or shy to engage in social activities (i.e., risk-taking) as their constant self-evaluation stops them from defying societal norms (Swami et al., 2021). In contrast, males who report feeling “about right” exert confidence in complying with societal ideals, and in turn engage in more socially oriented risk-taking (Swami et al., 2021).

Our findings are highly aligned with existing studies conducted in other contexts. Of 50 such studies reviewed during development of this study, most focused only on females and considered risk-taking behaviours in isolation. Past investigations have shown that females with alternate weight perception are prone to binge drinking (Raffoul et al., 2018b) and that current smoking behaviour is more likely among adolescents who perceive themselves as overweight, with higher effect sizes in females (Yoon and Bernell, 2016).

Major study strengths include the grounding of this analysis in theory, and use of a large and nationally representative sample. We considered the possible effect of sex in our analysis, whereas most existing studies focus exclusively on the experiences of females. Limitations of our study also warrant comment. First, our analysis involves a cross-sectional design which limits the ability to make causal inferences. Second, our analyses were also restricted to available indicators of risk-taking from a traditional general health survey. We acknowledge that other, more contemporary expressions of risk-taking do exist (Vente et al., 2020), which could lead to some misclassification of study outcomes. Third, the variable used in the 2018 HBSC provides a limited view of sex and gender and forced us to consider our questions with a view to the influence of biological sex only. This could have led to misclassification of at-risk groups. Furthermore, due to privacy issues, only those participants who self-identified as males or females could be included in sex-stratified analyses. Young people who do not identify as male or female may experience differential outcomes of weight perception (Kennis et al., 2022), so their omission is unfortunate but necessary. Fourth, sample size concerns among several racial and ethnic groups in the stratified data resulted in the need to amalgamate minority groups together into one category. We recognize that there are likely between-group differences in weight perception among minority groups, and though measuring these differences was not possible in this study, we encourage this as an aim for future works. Finally, stigma related to different body types may also contribute to misclassification (Brewis et al., 2016), as such stigmatization can lead to distress, impacting mental and physical health, and in some cases exacerbating health challenges to a greater level than the excess weight itself (Brewis et al., 2016). This stigma is not accounted for in the current analysis.

These findings have several important implications for prevention and clinical practice. To address such an important contemporary health issue, one must begin with the young people themselves, trying to change their attitudes, beliefs, how they think about their body, and what behaviours they subsequently choose to engage in (Morrongiello & Lasenby-Lessard, 2007). Interventions commonly consider weight perception and overt risk-taking as separate issues, yet there may be value in considering them together as common factors and experiences. Cluster-based risk-taking interventions have previously been demonstrated as more efficacious (Hale et al., 2014), and would likely be even more so if novel risk factors such as alternate weight perception are considered. Furthermore, our finding that males and females are differently affected by weight perception, and likely have different coping experiences, should be considered. Congruent with our finding that females with alternate weight perception are more likely to partake in risk-taking behaviours, which is often a group activity, females are also more prone to social influence and peer pressure (Dir et al., 2017). Therefore, programs that focus on social skills training, coping skills, and small group–based therapies are most beneficial in mitigating female substance use (Dir et al., 2017). Adding a cognitive behavioural therapy component (Lewis-Smith et al., 2019) to such programs to encourage development of positive self-image and confidence has the potential to create more efficacious programming that also addresses alternate weight perception as a risk factor (Jarry and Cash, 2011). As our finding among males suggested a null or even protective relationship between alternate weight perception and risk-taking behaviours, this paradoxical finding shows the need to consider sex when designing interventions based upon these identified correlations. Finally, in-depth research is required into the perceptions and behaviour of young people who identify as non-binary, as their experiences may be unique.

## Conclusion

This original analysis has demonstrated how weight perception is influenced by sex and then how it is associated with different levels of adolescent risk-taking. Alternate weight perception is very common among females and relates strongly and consistently with engagement in overt risk-taking. Addressing alternate weight perception as a potential risk factor for overt risk-taking in female adolescents appears to be a priority. Conversely, patterns of weight perception vary among males, with evidence that youth with an alternate weight perception tend to have lower engagement in risk behaviours. As males and females perceive and react to weight perception in different ways, clinical and health promotion strategies should be potentially developed and targeted differently to males and females with respect to these associations.

## Contributions to knowledge

What does this study add to existing knowledge?

- Few nationally representative Canadian data describe the presence and potential effects of alternate weight perception on the health behaviours of young Canadians.
- Existing studies primarily focus on female adolescents. This analysis examined the frequency of alternate weight perception among both adolescent males and adolescent females and its potential influence on engagement in risk-taking behaviours.
- Results show that alternate weight perception is common among adolescents, manifesting differently by sex. Females are prone to engagement in overt risk-taking (e.g., substance use) when experiencing alternate weight perception, whereas males who experience alternate weight perception are less likely to engage in risk-taking.

What are the key implications for public health policy and practice?

- Study findings provide new evidence to inform prevention efforts that are sensitive to the specific needs of groups of adolescents defined by sex.
- Males and females perceive and react to alternate weight perception in different ways; hence, health promotion strategies including associated policies should potentially be developed and targeted differently by sex. Females who identify as “too fat” are at the greatest risk for engagement in overt risk-taking and provide a natural focus for such countermeasures.
